# The Effects of Statins on Infections after Stroke or Transient Ischemic Attack: A Meta-Analysis

**DOI:** 10.1371/journal.pone.0130071

**Published:** 2015-07-09

**Authors:** Shao-Peng Lin, You-Ming Long, Xiao-Hui Chen

**Affiliations:** 1 Department of Emergency, the Second Affiliated Hospital of Guangzhou Medical University, 250# Changgang East Road, Guangzhou, 510260, Guangdong Province, China; 2 Department of Neurology, the Second Affiliated Hospital of Guangzhou Medical University, 250# Changgang East Road, Guangzhou, 510260, Guangdong Province, China; Medical University Innsbruck, AUSTRIA

## Abstract

**Background:**

Previous studies have reported that statins can prevent infections, and these findings were ascribed to the anti-inflammatory and immunomodulatory properties of statins. However, the effects of statins on the risk of infection after stroke or transient ischemic attack (TIA) remain controversial. The aim of this study was to evaluate the relationship between statins and the risk of infection after stroke or TIA by means of a meta-analysis.

**Methodology and Findings:**

Studies were found by searching major electronic databases using key terms and restricting the results to studies published in English language and human studies. Pooled odds ratio (OR) for the association between infection and statins were analyzed using Stata software. A total of five studies that included 8,791 stroke or TIA patients (3,269 patients in the statin use group and 5,522 in the placebo group) were eligible and abstracted. Pooled analysis demonstrated that statins did not significantly affect the incidence of infection after stroke or TIA compared with a placebo (OR 0.819, 95% CI 0.582–1.151, *I^2^* = 64.2%, *p*= 0.025). Sensitivity analyses showed that the removal of any single study did not significantly affect the pooled OR. Cumulative meta-analysis showed that the incidence of infection did not vary by publication year. No statistical evidence of publication bias was found among the studies selected, based on the results of Egger’s (*p* = 1.000) and Begg’s (*p* = 0.762) tests.

**Conclusions:**

This meta-analysis does not support the hypothesis that statins reduce the risk of infections in stroke or TIA patients.

## Introduction

Currently, HMG-CoA reductase inhibitors (statins) are widely used in the world, owing to their anti-atherosclerotic benefits [1]. In addition to reducing serum cholesterol levels, statins have anti-inflammatory and immunomodulatory properties, as shown by in vitro and animal experiments [2–5], suggesting that they may have a beneficial role in infection. In recent years, statins were found to have additional beneficial effects on the prevention and treatment of infections in some diseases, such as ischemic heart disease, cerebrovascular accident, or peripheral vascular disease [6– 8]. However, other large-scale studies have found that statin treatment was associated with an unexpected increase in the incidence of infection in those patients [9–11]. These findings are conflicting and current data regarding the role of statins in patients with atherosclerotic disease is scarce.

The incidence of post-stroke/transient ischemic attack (TIA) infection is as high as 25–30%, with pneumonia and urinary tract infections being particularly common [12–14]. A number of infections have been shown to worsen the clinical course and outcome after acute stroke and TIA [15–17]. Current guidelines recommend the use of statins during initial hospitalization for stroke or TIA to prevent recurrent ischemia [18]. However, few randomized controlled trials have prospectively investigated the effects of treatment with statins on infections in patients diagnosed with stroke or TIA. A recent meta-analysis failed to show a significant effect of statins on infections not restricted to stroke and TIA patients [19]. Because infectious complications occur frequently after stroke and TIA, it is important to review the role of statins in preventing infections in stroke and TIA patients, in particular. Given that the effects of statins in infection after stroke or TIA are still unclear, we conducted a meta-analysis to determine whether an association between the use of statins after stroke or TIA and the risk of infection exists.

## Methods

### Data sources

We performed a systematic search of three major electronic databases (PubMed, EMBASE, and the Cochrane Library) for literature published from the inception of each database through July 2014. The following key words were used in our search strategies: (“statins” or “fluvastatin” or “simvastatin” or “atorvastatin” or “rosuvastatin” or “lovastatin” or “pravastatin” or “hydroxymethylglutaryl-coA reductase inhibitors”)[20] and (“infection” or “bacteremia” or “pneumonia” or “sepsis”) and (“stroke” or “brain ischemic” or “transient brain ischemia” or “cerebral arterial disease” or “CVA” or “non-ischemic stroke” or “ischemic stroke” or “cerebrovascular accident” or “intracranial artery disease”) [21]. The searches were limited to “human studies” and “English language”. The bibliographies of retrieved articles were checked by the authors to identify other potentially eligible studies.

### Selection of studies and quality assessment

Randomized single or double-blind trials, placebo-controlled studies, observational cohort studies and case-controlled studies were included. Participants in the studies were patients who had suffered a stroke or TIA. Animal experimental studies were excluded. Exposure was defined as the use of statins for any reason. The outcome was defined as infection from any cause.

Two authors (Shao-Peng Lin and You-Ming Long) independently screened each of the potential trials to determine whether it should be included. If the two authors had different opinions or were uncertain, the third author (Xiao-Hui Chen) was consulted to make a decision. Characteristics, including the source, publication date, country, design, sample size, and type of infection were collected for each publication. The quality of the studies was evaluated by using the Newcastle—Ottawa Scale [22]. The quality score was calculated on the basis of three items: the selection of the study groups (0–4 points), the comparability of the study groups (0–2 points), and the assessment of the outcome (0–3 points). Higher scores represented studies of better quality.

**Fig 1:**
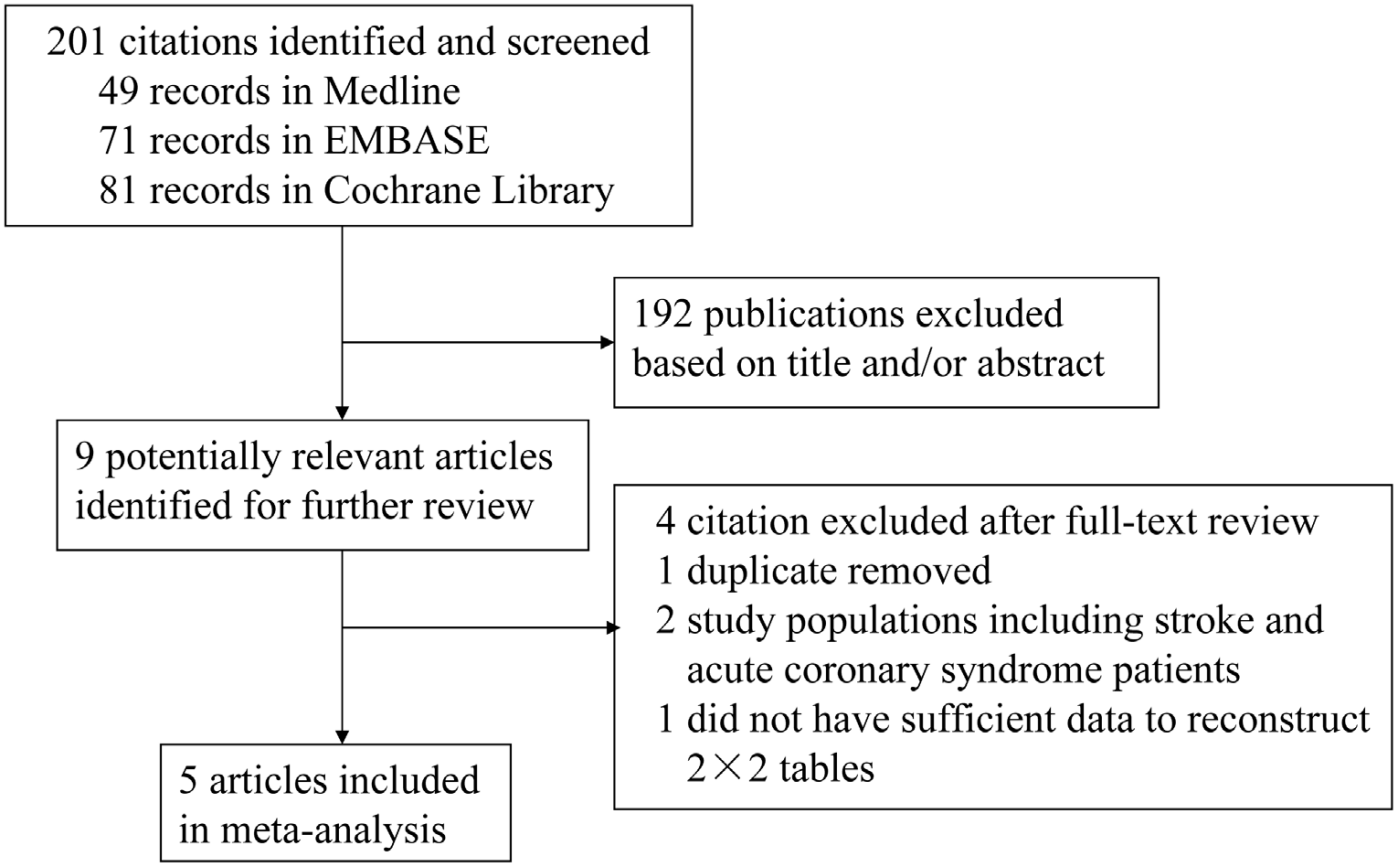
PRISMA flow chart of study identification and inclusion.

The electronic database search identified 201 potentially eligible articles. After initial screening based on the title and/or abstract, 192 articles were excluded, and nine articles were chosen for full evaluation. Of those, two articles [23, 24] were by the same authors at the same research institution, so they were considered as one study. We chose the study that contained the more recent and complete data on the association between statins and infection after stroke for analysis in the meta-analysis. Two studies were excluded because the study population included stroke and acute coronary syndrome patients [9, 25]. One study that did not provide sufficient data to reconstruct 2 × 2 tables was also excluded [11]. Eventually, five reports were included in this meta-analysis (Fig 1).

### Statistical analysis

We assessed the heterogeneity across studies using the *I* ^2^ statistic, which quantifies the percentage of variability that can be attributed to between-study differences [26]. A random effects model was used to calculate pooled odds ratio (OR) for all analyses using the metan command in Stata. Sensitivity analysis was carried out by recalculating the summary statistics after removing individual studies from the analysis to assess the influence of each study. Cumulative meta-analysis was used to explore any significant variation in the incidence of infection by publication year. Begg’s test and Egger’s test were performed to evaluate the possibility of publication bias, and a funnel plot was generated to visualize the potential for asymmetry [27]. All statistical analyses were performed using Stata intercooled version 12 (StataCorp, College Station, TX, USA). The meta-analysis was conducted in accordance with the relevant criteria given by the PRISMA (Preferred Reporting Items for Systematic Reviews and Meta-Analyses) statement [28].

## Results

### Characteristics of the included studies

A total of 201 studies were identified by our initial search strategy (Fig 1). After screening the titles and abstracts, 192 publications were excluded, and nine articles were selected for full-text review. Four articles were excluded after full-text review, leaving a total of five studies [7, 10, 23, 29, 30] that met the inclusion criteria for this meta-analysis.

The characteristics of the selected studies are shown in Table 1. In the five trials, one [10] was conducted primarily in the United States and Europe, and four [7, 23, 29, 30] were conducted in European countries. The number of participants ranged from 112 in the study by Becker et al.[10] to 4,731 in the study by Amarenco et al.[29]. One study was a randomized controlled trial (RCT)[29], and four were prospective cohort studies [7, 10, 23, 30]. A total of 8,791 patients (3,269 with statin treatment and 5,522 without statin treatment) were included in the five studies eligible to be included in the meta-analysis. Two studies reported the effect of statins on pneumonia [7, 30]. Two studies reported the effect of statins on pneumonia and other types of infections [10, 23] and one study did not mention the type of infection [29]. All studies were of high quality according to an analysis using the Newcastle—Ottawa Scale.

**Table 1:** Characteristics of the included studies. RCT, randomized controlled trial; PC, prospective cohort study; tPA, tissue plasminogen activator; NR, not recorded.

**A**									
**Source**	**Country**	**Year**	**Design**	**Study population**	**Mean age (y)**	**Male sex (%)**	**Patients (n)**		
							**Total**	**Statins**	**Non-statin**
Amarenco P et al.[29]	Multinational cooperation	2006	RCT	Stroke and transient ischemic attack	63	60	4731	2365	2366
Arboix A et al.[30]	Spain	2010	PC	First-ever ischemic stroke	NR	47	2082	381	1701
Scheitz JF et al.[7]	Germany	2012	PC	Acute ischemic stroke receiving tPA	NR	50	481	83	398
Becker K et al.[10]	American	2013	PC	Acute ischemic stroke	57	65	112	79	33
Rodríguez-Sanz A et al.[23]	Spain	2013	PC	Acute ischemic stroke	68	60	1385	361	1024
**B**									
**Source**	**Comparison**	**Follow-up**		**Type of infection**	**Infection rates (%)**	**Effect of statins on infection**			**Quality score**
Amarenco P et al.[29]	Statin use vs non	5 years		NR	18	Neutral			8
Arboix A et al.[30]	Statin use vs non	During the hospital stay		Pneumonia	12	Beneficial			7
Scheitz JF et al.[7]	Statin use vs non	During the hospital stay		Pneumonia	9	Beneficial			7
Becker K et al.[10]	Statin use vs non	15 days after stroke onset		Pneumonia, urinary tract infections and others	26	Harmful			7
Rodríguez-Sanz A et al.[23]	Statin use vs non	During the hospital stay		Pneumonia, urinary tract infections and sepsis	9	Neutral			7

Two of the five studies [23, 29], which accounted for more than 58% of the pooled patient sample, found no effect of statins in terms of the incidence of infection. Two studies [7, 30] demonstrated a decreased incidence of infection in patients taking statins, while one study [10] reported an increased incidence of infection in patients taking statins.

### The influence of statins on infection after stroke or TIA

**Fig 2:**
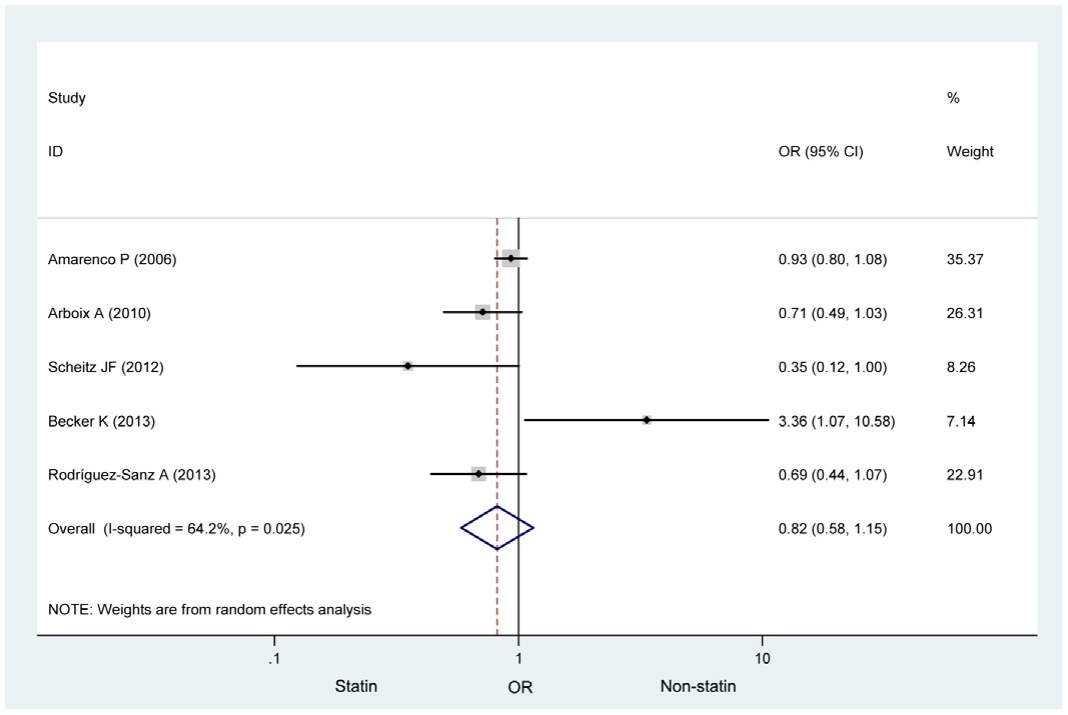
The association between statins and infection after stroke or TIA.

**Fig 3:**
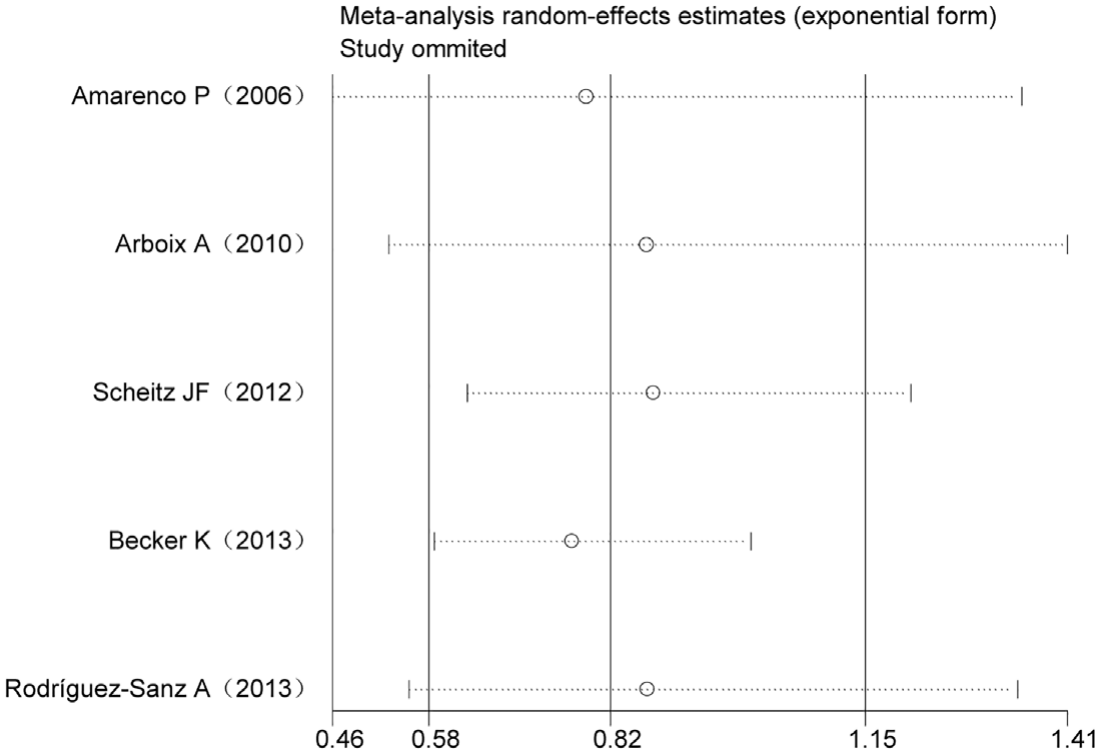
Sensitivity analyses for the five included studies (Each small circle indicates mean differences. The intervals between the two short lines around each circle indicate the 95% CI of each study).

**Fig 4:**
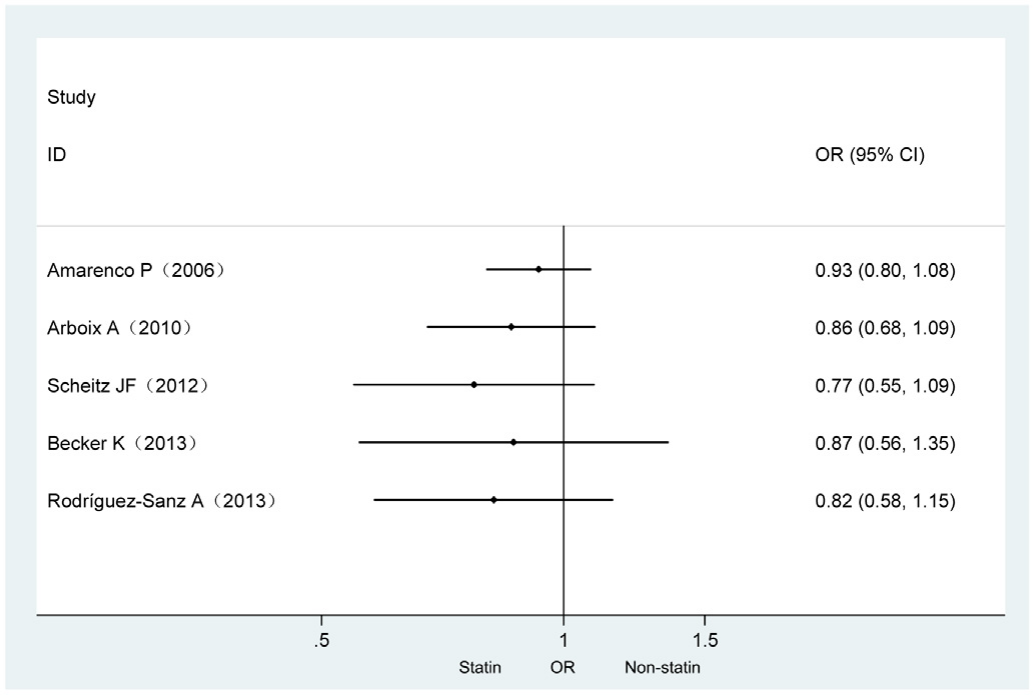
Cumulative meta-analysis of the effect of statins on infection after stroke or TIA(Note: weights are from random effects analysis).

**Fig 5:**
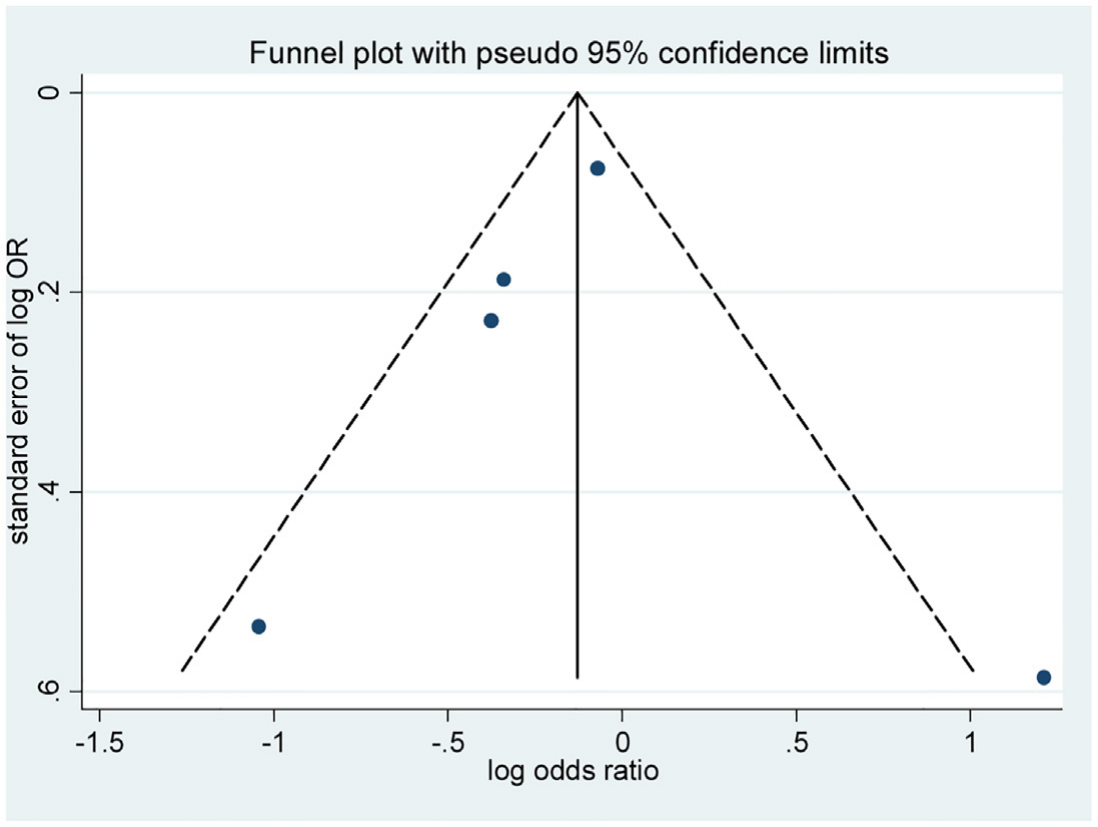
Begg’s funnel plot for the analysis of publication bias in studies on the incidence of infection, including all five studies comparing patients exposed and unexposed to statins.

The heterogeneity among the studies was statistically significant (*I* ^2^ = 64.2%, *p* = 0.025). Thus, the primary meta-analysis was assessed using a random effects model. The pooled analysis indicated that statins did not significantly affect the incidence of infection after stroke or TIA compared to a placebo (OR 0.819, 95% CI 0.582–1.151; Fig 2). Sensitivity analyses showed that the removal of any one study did not influence the pooled OR (Fig 3). Cumulative meta-analysis was used to examine the possibility that the incidence of infection in the eligible studies could have varied with the publication year. The cumulative meta-analysis demonstrated that the incidence of infection was stable across publication years (Fig 4). There was no statistical evidence of publication bias among the studies, based on the results of Egger’s test and Begg’s test (*p* = 1.000 and *p* = 0.762, respectively). The funnel plot was examined visually (Fig 5).

## Discussion

We conducted a meta-analysis to evaluate the efficacy and safety of statins in stroke or TIA patients. This quantitative analysis found that statins have no effect on the incidence of infection after stroke or TIA.

At present, the effects of statin treatment on the risk of infection after stroke or TIA remains controversial. Some studies have suggested that statin therapy may decrease the risk of infection. Arboix et al.[30] found that the use of statins had a beneficial effect on the prevention of pneumonia and reduced the rate of stroke-associated pneumonia during hospitalization. Although the pharmacological mechanisms underlying this effect on infection remain unclear, it may be explained by the anti-inflammatory properties of statins [31]. There are multiple potential anti-inflammatory mechanisms by which statins might decrease the risk of infection. First, statins could optimize the nitric oxide availability in acute inflammatory states. Second, statins might also suppress endothelial dysfunction due to inflammation. Third, statins were shown to decrease inflammatory biomarkers such as C-reactive protein, providing further evidence of their general anti-inflammatory activity [32, 33]. Scheitz et al.[7] found that statin therapy in ischemic stroke patients treated with thrombolytic agents also reduced the risk of poststroke infection, suggesting a possible interaction between tissue plasminogen activator (tPA) and statins. Animal experiments have shown that statins may enhance the thrombolytic effect of tPA and that effective thrombolysis can decrease systemic inflammatory responses [34, 35]. Therefore, statin treatment may contribute to the prevention of pneumonia by enhancing the response to tPA.

On the other hand, some studies have reported a detrimental effect of statins. Two clinical pilot trials demonstrated a higher frequency of infection in patients treated with statins in the acute stage of ischemic stroke [10, 11]. The most plausible explanation for this finding is the immunosuppressive effect of statins. Statins inhibit the acquired immune response by suppressing antigen presentation, reducing T-cell function, and decreasing T-cell proliferation [36]. Therefore, statins might increase the risk of infection by interfering with initiation of the immune response.

Other studies have failed to find an association between statin treatment and infection in stroke or TIA patients. A large randomized controlled trial [29] and a prospective cohort study [23] proposed a possible relationship between statin treatment and the development of infection but found no significant correlation between statin treatment and the risk of infection.

Because statins have potent immunomodulatory properties, and the safety of these agents after stroke or TIA has not been clearly established, we performed a meta-analysis to evaluate the relationship between the use of statins after stroke or TIA and the risk of infection. Our quantitative analysis found that statins neither significantly increase nor decrease the incidence of infection after stroke or TIA. The group of van den Hoek et al.[19] report an association between statins and infections not restricted to stroke or TIA, but they also did not find any association between statins and infection in randomized clinical trials. Frequent infectious complications after stroke or TIA and post-stroke/TIA immunosuppression suggest that it is important to review the effects of statins in stroke and TIA in particular [37–39]. Our results show no evidence that statins have an effect on the risk of infection after stroke or TIA; this finding does not support the previous hypothesis that statins have an effect on the risk of infection after stroke or TIA. Several factors may explain the inconsistent results of these studies. First, these reports considered only whether high doses of statins can effectively prevent and treat infections [4, 40]. However, these doses exceed the single dose of statins given to stroke or TIA patients by far. Moreover, statin users often have lighter disease states, better overall function and higher socioeconomic status than non-users, resulting in the so-called healthy-user effect [41]. In summary, the finding in previous observational studies that statins are effective in the prevention of infections may result from biased sample selection, which is inherent in observational research [19]. Therefore, it is necessary to conduct further research to determine whether statins have beneficial effects on the risk of acquiring an infection after stroke or TIA.

Our study has several strengths. To our knowledge, this meta-analysis is the first study to analyze the role of statins in the development of infectious complications after acute stroke or TIA. We tried to minimize any bias by retrieving articles from three major electronic databases, and statistical tests found no evidence of publication bias among the studies examined. Furthermore, removal of any one study did not significantly influence the pooled OR. Finally, cumulative meta-analysis showed that the incidence of infection was stable across the publication years.

There are several limitations to our meta-analysis. One potential limitation is the different types of infection that were considered in the studies examined. Some studies took only respiratory infections into account and did not take infections of other systems into account [7, 30]. Another limitation is the heterogeneity of follow-up time, which may be relevant given that the effect of statins on infection is likely to be dose- and time-dependent [40, 42]. Because only five studies were included in this meta-analysis, we could not perform subgroup analyses for infection type, statin dose and follow-up time. Lacking of a large number of studies limited our further evaluation of potential heterogeneity.

In conclusion, our meta-analysis suggests that the possible immunosuppressive effect of statins is not significant in stroke or TIA patients. Treatment with statins neither increased nor decreased the risk of infection in these patients. However, the results of this meta-analysis should be interpreted with caution because of the heterogeneity among the study designs of the studies that were examined. We cannot rule out the possibility that statins may have an effect on infection in some categories of stroke. Further research should focus on confirming the influence of statins in acute ischemic stroke patients who receive thrombolytic therapy. This combination may have important effects on the prognosis of patients suffering from ischemic stroke.
